# Protein Clearance Mechanisms of Alpha-Synuclein and Amyloid-Beta in Lewy Body Disorders

**DOI:** 10.1155/2012/391438

**Published:** 2012-10-22

**Authors:** Michela Deleidi, Walter Maetzler

**Affiliations:** ^1^German Center for Neurodegenerative Diseases (DZNE), University of Tuebingen, 72076 Tuebingen, Germany; ^2^Center of Neurology, Department of Neurodegeneration, Hertie Institute for Clinical Brain Research, University of Tuebingen, Hoppe Seyler-Straße 3, 72076 Tuebingen, Germany

## Abstract

Protein clearance is critical for the maintenance of the integrity of neuronal cells, and there is accumulating evidence that in most—if not all—neurodegenerative disorders, impaired protein clearance fundamentally contributes to functional and structural alterations eventually leading to clinical symptoms. Dysfunction of protein clearance leads to intra- and extraneuronal accumulation of misfolded proteins and aggregates. The pathological hallmark of Lewy body disorders (LBDs) is the abnormal accumulation of misfolded proteins such as alpha-synuclein (Asyn) and amyloid-beta (Abeta) in a specific subset of neurons, which in turn has been related to deficits in protein clearance. In this paper we will highlight common intraneuronal (including autophagy and unfolded protein stress response) and extraneuronal (including interaction of neurons with astrocytes and microglia, phagocytic clearance, autoimmunity, cerebrospinal fluid transport, and transport across the blood-brain barrier) protein clearance mechanisms, which may be altered across the spectrum of LBDs. A better understanding of the pathways underlying protein clearance—in particular of Asyn and Abeta—in LBDs may result in the identification of novel biomarkers for disease onset and progression and of new therapeutic targets.

## 1. Introduction

Lewy body disorders (LBDs) is an umbrella term that includes diseases with alpha-synuclein (Asyn) aggregates as fibrils in Lewy bodies (LBs) and Lewy neurites. Several lines of evidence support a pathogenic role of misfolded Asyn in LBDs [1–3]. Parkinson's disease (PD) without dementia (PDND) is the most common subtype of LBDs, followed by PD with dementia (PDD) and dementia with Lewy bodies (DLBs) [4, 5]. Like in PD, the core feature of PDD is a diagnosis of PD according to the UK Brain Bank Society criteria [6] but also includes cognitive symptoms severe enough to fulfil dementia criteria at least one year after PD diagnosis with insidious onset, slow progression, and impairment in more than one cognitive domain [7]. DLB is the second most prevalent neurodegenerative dementia after Alzheimer's disease (AD). Clinical diagnosis is based on the presence of a dementia syndrome, accompanied by at least two out of the three following symptoms: fluctuating cognition with pronounced variations in attention and alertness, visual hallucinations, and Parkinsonism [8]. In contrast to PDD, the onset of dementia in DLB is before or within one year of any Parkinsonism.

About one-fourth of LBDs patients show cortical amyloid-beta (Abeta) deposition, with the highest proportion in DLB subjects, followed by PDD subjects [9–14]. According to clinical, neuroimaging, and neuropathological data, co-occurrence of Asyn and Abeta is regularly associated with dementia in LBDs [13–15]. 

Based on the central role that Asyn and Abeta play in the pathogenesis of LBDs and the increasing body of literature pointing to defective clearance of misfolded proteins as a key mechanism to the pathogenesis of LBDs, this paper aims at providing a condensed review of this latter topic. Although not exhaustive, it may provide a basic understanding of such mechanisms eventually contributing to the development of novel disease biomarkers (which are currently not available [16, 17]) and neuromodulatory treatment strategies for these still incurable and chronic diseases. 

## 2. General Mechanisms of Protein Clearance

The main *(intraneuronal)* pathways for the degradation and recycling of proteins are the ubiquitin/proteasome system (UPS) and the autophagy-lysosomal pathway (macroautophagy, microautophagy, and chaperone-mediated autophagy (CMA)). 

The UPS degrades short-lived nuclear and cytosolic proteins or misfolded proteins in the endoplasmic reticulum [18]. It plays a key role in signal transduction, cell cycle progression, apoptosis, and cellular differentiation and has been implicated in several human diseases, including neurodegenerative diseases, cancer, inflammation, and autoimmunity [18–20]. 

Autophagy is a process involving the degradation of components inside lysosomes [21, 22]. It has a variety of physiological and pathophysiological roles in protein and organelle clearance, development, defence against microorganisms, cell death, and antigen presentation [23]. In macroautophagy, organelles and macromolecular components are first surrounded by a double membrane, defined as the autophagosome or autophagic vacuole (AV), which then fuses with lysosomes to form autolysosomes. In microautophagy, the transfer of cytosolic components the lysosomal compartment happens by direct invagination of the lysosomal membrane without prior sequestration into the autophagosome. Finally, in CMA, individual proteins are targeted to lysosomes by the binding of a hsc70-containing chaperone/cochaperone complex. 

All these pathways are observable in neurons, and both impairment and excessive activation of these pathways are linked to neurodegenerative processes [24].

(Extraneuronal) clearance pathways include interaction of neurons with astro- and microglia and with infiltrating macrophages. Microglial cells are considered the professional phagocytes in the brain. However, other populations of cells may also act as phagocytes, including astrocytes, neural stem cells, and neurons [25–28]. There is no clear evidence for a defective protein clearance by the brain innate immune system as a primary pathogenetic event in neurodegenerative diseases. However, phagocytosis of misfolded proteins by astro- and microglia triggers the release of proinflammatory cytokines and chemokines and reactive oxygen/nitrogen species, which may, under pathological conditions, further promote neuronal dysfunction and degeneration [29, 30]. 

The adaptive immune system is also involved in the clearance of misfolded proteins in the brain. Naturally occurring autoantibodies are detectable in body fluids of healthy controls and in patients with neurodegenerative disorders [31–33]. Finally, protein transport from the parenchyma to the cerebrospinal fluid (CSF) and across the blood-brain barrier are relevant clearance mechanisms and have been shown to be affected in LBDs. 

**Figure 1:**
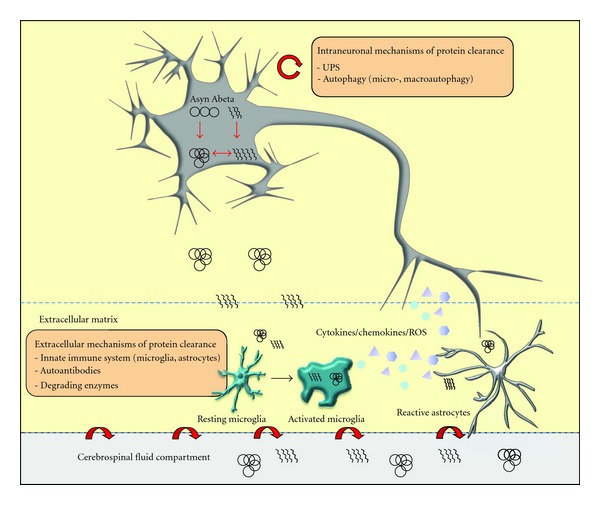
Intra- and extraneuronal mechanisms of protein clearance. The main intracellular pathways for the degradation and recycling of proteins are the ubiquitin/proteasome system (UPS) and the autophagy-lysosomal pathway (microautophagy, macroautophagy, and chaperone-mediated autophagy (CMA)). Extracellular clearance pathways include interaction of neurons with astro- and microglia, and with infiltrating macrophages, autoantibodies, and protein transport from the parenchyma to the cerebrospinal fluid and across the blood-brain barrier. The engulfment of misfolded proteins by astro- and microglia triggers the release of proinflammatory cytokines and chemokines as well as reactive oxygen/nitrogen species, which may, under pathological conditions, further promote neuronal dysfunction and degeneration.

Intra- and extraneuronal clearance mechanisms closely interact: as an example, protein aggregates can stimulate the cell surface innate immune receptors, initiating intracellular signaling cascades that, in turn, stimulate phagocytosis. An overview of how these mechanisms may interact is schematically illustrated in Figure 1.

In the following, all these mechanisms are discussed in more detail with special emphasis on Asyn and Abeta clearance.

## 3. Asyn Clearance 

### 3.1. Intraneuronal Mechanisms

Abnormal deposition of Asyn occurs early in the disease process of LBDs and may follow an ascending pattern in most of the cases, starting from lower brainstem areas and then affecting limbic and cortical areas [34]. Deposition may start even earlier in the autonomic peripheral nervous system [35, 36]. The mechanisms responsible for Asyn degradation have been controversial, but it appears that, under normal conditions, Asyn is degraded by both the UPS and the autophagy-lysosomal pathway [37–39], whereas the autophagy-lysosomal pathway mediates clearance of accumulated and aggregated Asyn [38]. In agreement with this observation, activation of autophagy leads to increased wild-type Asyn clearance and neuroprotection [37, 40]. Normal Asyn binds to the CMA-specific receptor LAMP-2A on the lysosomal membrane and is subsequently degraded by CMA. However, mutant forms of Asyn (A53T and A30P) and Asyn modified by dopamine tightly bind to the CMA receptors on the lysosomal membrane and inhibit both their own degradation and that of other CMA substrates [38, 41]. The dysfunction of CMA triggers neuronal dysfunction and increases vulnerability to stress. Interestingly, both mutant and wild-type Asyn can decrease proteasomal activity and increase vulnerability to neurodegeneration, leading to a vicious cycle where an increased amount of intraneuronal Asyn can block its clearance by itself [38, 42–46]. 

Several studies provide evidence for impaired autophagy-mediated clearance mechanisms in PD. The main proteins involved in CMA (Lamp2a and Hsc70) are decreased in the SN and amygdala from PD patients [46]. Microtubule-associated protein 1A/1B-light chain 3 (LC3, a marker for autophagic vacuoles) colocalizes with Asyn in most LBs and Lewy neurites [46]. 

Genetic mutations leading to Parkinsonism support the hypothesis that defective clearance mechanisms are centrally involved also in idiopathic forms of LBDs. Interaction between PINK1 and Parkin can modulate mitophagy [47, 48]. PINK1 itself directly activates autophagy and interacts with autophagic proteins such as Beclin1 [49]. A reduced clearance of mitochondria was also demonstrated in cells lacking DJ-1 which is another protein associated with recessive forms of Parkinsonism [50]. Mutations in the *LRRK2* gene, the most common form of late onset autosomal dominant Parkinsonism, may also cause neuronal cell death via impairment of protein degradation pathways as they influence the autophagy-lysosomal pathway, leading to Asyn accumulation and aggregation [51, 52]. Heterozygous mutations in the gene encoding the lysosomal enzyme glucocerebrosidase (GBA) convey an approximately 5-fold risk for PD. These mutations are associated with lysosomal dysfunction and influence binding of Asyn to its specific receptor at the lysosome membrane: a recent study demonstrated that the accumulation of the GBA substrate glucosylceramide stabilizes Asyn soluble oligomers which, in turn, can inhibit normal GBA lysosomal activity. This creates a positive feedback loop that could directly contribute to the neurodegenerative process [53]. The heterozygous *GBA* mutation also seems to directly influence fatty acid metabolism: PD patients with these mutations have lower CSF levels of palmitoleic, oleic, linoleic, arachidonic, eicosapentaenoic, and docosahexaenoic acids compared with both idiopathic PD patients and controls [54]. 

### 3.2. Extraneuronal Mechanisms (Mainly)

Although Asyn is a cytosolic protein, a low quantity of the protein is secreted via vesicle exocytosis and is then present in biological fluids including plasma and CSF [55–57]. The mechanisms responsible for the extraneuronal clearance of Asyn are not entirely clear; however, there is increasing evidence that adjacent cells such as astrocytes and microglia as well as the adaptive immune system and local protein transport mechanisms are crucially involved. 

Loss of midbrain dopaminergic neurons and striatal degeneration can be preceded by neuroinflammation marked by activated microglia and an increase in proinflammatory cytokines and chemokines [29, 30, 58–62]. Astrocytes are also involved in the initiation and progression of the disease [63]. A recent study showed that astrocytic Asyn initiates noncell autonomous killing of neurons [64]. Indeed, extraneuronal forms of Asyn can activate glial cells and trigger inflammation and subsequent release of proinflammatory molecules, a common pathological hallmark of LBDs [60, 65]. Several studies have reported Asyn-containing inclusions in astroglia of PD and DLB patients [66], and phagocytic microglial cells are very efficient scavengers of extraneuronal Asyn aggregates [63, 67].

Results of recent GWAS studies argue for the relevance of the immune system, in the pathophysiology of LBDs, as variations in the *human leukocyte antigen (HLA)* region were associated with occurrence of LBDs [68, 69]. This may also be mediated through microglia as these cells are capable of presenting antigens to lymphocytes [70] via the HLA domain. Activation of HLA-positive microglia is observable in affected brain regions of PD patients [71]. Asyn autoantibodies are more prevalent in sera of PD patients than in controls [31]. These results were basically confirmed by a recent study where serum Asyn autoantibody levels were higher in demented LBDs patients than in controls [72]. 

An overload of local Asyn can also occur due to defective transport mechanisms of Asyn from the neuron to the CSF. Most studies investigating CSF total Asyn levels in PD showed that these levels are reduced in the CSF of PD patients compared to controls, whereas oligomeric Asyn levels seem to be higher in PD compared to control CSF [57, 73–77]. A recent study showed that CSF levels of phosphorylated Asyn correlate weakly with PD severity and, if corrected for total Asyn, contributed to the differential diagnosis between PD, multiple system atrophy (MSA), and progressive supranuclear palsy (PSP) [78]. However, it is neither clear to date how Asyn is transported from the parenchyma to the CSF, nor why total Asyn is reduced and pathological forms of Asyn are elevated in the CSF of PD patients. 

## 4. Abeta Clearance: Lessons from Alzheimer's Disease Research

Comparable to LBDs pathophysiology, in AD, only some rare genetic mutations lead to increased APP expression or to changes in Abeta stability or aggregation [79]. The common late onset sporadic AD seems to be far better explained by impaired Abeta clearance mechanisms [80]. Thus, assuming that similar mechanisms may occur in (late onset) AD and LBDs and that Abeta pathology is a pathophysiologically relevant feature of LBDs, a discussion of (ineffective) Abeta clearance mechanisms as they occur in AD may substantially contribute to our understanding of how Abeta may (or may not) be cleared in LBDs. 

### 4.1. Intraneuronal Mechanisms

There is convincing evidence from electron microscopy studies [81] that the autophagy-lysosomal pathway is involved in the pathogenesis of AD. AVs which are the major reservoir of intraneuronal Abeta are abundant in affected neurons, especially in those with neurofibrillary tangles [82]. These studies argue for an impaired maturation of autophagolysosomes and impaired intraneuronal retrograde transport in AD. Defective clearance of Abeta-generating AVs may result in Abeta accumulation [83, 84]. Several further findings corroborate the hypothesis that impaired autophagy plays a key role in the pathogenesis of neuronal degeneration in AD. Beclin1 is decreased in AD brains, and decreased neuronal autophagy and subsequent lysosomal dysfunction and neurodegeneration are observed in mice carrying a heterozygous deletion of Beclin1 [85]. Presenilin 1 (PS1) is essential for maturation of the lysosomal proton pump and affects autophagocytosis and protein turnover [86].

### 4.2. Extraneuronal Clearance Mechanisms (Mainly)

Abeta concentration is tightly regulated by amyloid-degrading proteolytic enzymes and perivascular drainage [87–89]. Neprilysin (NEP) is an Abeta-degrading protein found at presynaptic terminals and in body fluids [90, 91]. It is a preferentially membrane-bound, presynaptically located protein with an extracellular catalytic site which can degrade Abeta [90, 91]. A soluble form of NEP is detectable in body fluids such as blood and CSF, emanating from a slow release from the membranes [92]. Most interestingly, reduced CSF NEP activity levels have been shown to occur in early AD [93, 94]. 

Another protein involved in defective clearance mechanisms in AD is cystatin C. Neurons, among other cells, are able to produce and secrete this protein [95]. Fourfold higher levels of cystatin C in the CSF than in blood [96] indicate a relevant role of the protein in CNS pathways. Cystatin C binds monomeric Abeta and carries soluble Abeta [97, 98]. There is evidence that AD patients have reduced CSF cystatin C levels [99, 100]. This makes it intriguing to hypothesize that a deficient Abeta-binding capacity, as induced by a lack of (functional) cystatin C, may contribute to the amyloidogenic process in AD [99]. Indeed, increased expression of this protein has been shown to reduce parenchymal Abeta load in mouse models of AD [101, 102]. Of note, the *BB* genotype of the cystatin C-encoding gene—which leads to reduced cystatin C secretion from the neuron to the extracellular space [103, 104]—conveys susceptibility to AD [105]. 

Inflammatory reactions, characterized by activated microglia and astrocytes surrounding amyloid deposits, are intimately associated with the onset and progress of AD [106–108]. Reactive astrocytes with Abeta-positive granules are found in close proximity to amyloid plaques. Human astrocytes express scavenger receptors and several Abeta-degrading enzymes such as NEP, insulin-degrading enzyme (IDE), endothelin-converting enzyme (ECE), angiotensin-converting enzyme (ACE), plasminogen activators, and the matrix metalloproteinases-9 and -2 (MMP-9, MMP-2) [109–112]. These findings suggest an important role of astrocytes in Abeta clearance.

Clinicopathological studies suggest that microglial activation is an early event in AD pathology [113, 114]. Activated microglia surround amyloid fibril deposits, and postmortem studies have shown significant amounts of Abeta in microglial cells of AD patients treated with immunization therapy [115, 116]. Microglia express toll-like receptors (TLRs), a family of highly conserved molecules that recognize pathogen-associated molecular patterns, including both exogenous and endogenous ligands [117]. TLR2 and TLR4 have been associated with the removal of Abeta, indicating that the innate immune system plays a key role in preventing the brain from Abeta deposits [118–120]. 

In addition, the adaptive immune system is obviously involved in CNS parenchyma clearance mechanisms. Naturally occurring antibodies directed against Abeta have been detected in the CSF and plasma of patients with AD and healthy control subjects. Some studies have shown reduced CSF levels of anti-Abeta antibodies in patients with AD compared with healthy control subjects [32, 121] and in individuals at increased risk for AD [122]. Another study reported that a subset of conformation-specific, cross-reactive antibodies that may protect against amyloidogenic toxic peptides are reduced in AD patients [123]. As a consequence of these findings, a number of phase II and III clinical trials are currently under way to test the effect of such autoantibodies in AD patients [124]. First results are promising: a recent study using carbon 11-labeled Pittsburgh Compound B ([11C]PiB) positron emission tomography (PET) has shown that passive immunization can reduce the level of brain amyloid *in vivo* after 18 months of antibody treatment [125].

## 5. Abeta Clearance in Lewy Body Disorders

### 5.1. Intraneuronal Mechanisms

To the best of our knowledge there is no study available that investigated intraneuronal mechanisms of Abeta clearance in LBDs. There is however indirect evidence that deficits of (intraneuronal) defence mechanisms against Abeta toxicity may exist, at least in demented LBDs patients. We recently showed that CSF levels of uric acid, an antioxidant detectable in neurons and associated with PD progression, were significantly lower in demented than in nondemented LBDs patients. In addition, these levels correlated positively with CSF Abeta42 levels, with highest correlation values in controls and lowest in demented LBDs patients [126]. In the light of the recent finding that CSF Abeta levels increase within hours after trauma and thus reflect a sufficient and fast response to neuronal stress [127], a weak correlation of CSF Abeta42 with uric acid may indicate deficits in this repair mechanism. 

### 5.2. Extraneuronal Mechanisms (Mainly)

Lowered CSF NEP activity levels have been found in demented LBDs patients, compared to nondemented LBDs patients and controls [128]. In addition, CSF NEP activity levels correlated positively with CSF Abeta42. These data argue for a role of NEP in the pathophysiology of cognitive decline in LBDs. 

Also cystatin C seems to be relevantly involved in Abeta-associated cognitive decline in LBDs. Our group investigated CSF and serum levels in LBDs patients [129] and found lower CSF cystatin C levels in demented LBDs patients compared to PDND and controls. In additions, these levels correlated positively with age at onset of dementia but not with parameters associated with Parkinsonism. Notably, the correlation between CSF cystatin C and CSF Abeta42 levels was highly significant in nondemented individuals, but not significant in demented patients. This indicates that cystatin C-related Abeta transport from the neuron to the CSF is impaired in demented LBDs patients. This hypothesis is corroborated by genetic results [129]. The risk genotype of the *CST3* gene, *BB*, was detectable only in demented LBDs patients and was associated with low CSF cystatin C levels.

The role of the innate immune system in Abeta clearance in LBDs is not well understood although neuroinflammatory reactions are a common finding in LBDs and are considered to play a key role in the neurodegenerative process [63]. There is a tight association of microglia with degenerating LB-containing neurons [130]. Activated microglia is associated with Asyn-positive oligodendrocytes in MSA patients and in an animal model of this disease [131, 132]. Astrocytic abnormalities also occur [133, 134]. 

The adaptive immune system is also involved in Abeta-associated mechanisms in LBDs. Autoantibodies against Abeta were elevated in serum and CSF of demented LBDs patients, compared to controls and were even higher than in other forms of dementia such vascular dementia [72]. Still, many questions remain about the contribution of the immune system, such as microglia, macrophages, and T cells but also other immune cells, to clearance of misfolded proteins in the CNS.

## 6. Conclusion

Although many questions remain open, recent literature suggests that impairment of protein clearance is one of the key factors mediating the degeneration of vulnerable neuronal populations in LBDs. Both intra- and extraneuronal clearance mechanisms are impaired in LBDs. An improved understanding of such pathways can provide the basis for new developments in the biomarker era and, ultimately, contribute to the development of neuromodulatory or even causal treatment strategies.
